# Structure of the competence pilus major pilin ComGC in *Streptococcus pneumoniae*

**DOI:** 10.1074/jbc.M117.787671

**Published:** 2017-06-28

**Authors:** Sandra Muschiol, Simon Erlendsson, Marie-Stephanie Aschtgen, Vitor Oliveira, Peter Schmieder, Casper de Lichtenberg, Kaare Teilum, Thomas Boesen, Umit Akbey, Birgitta Henriques-Normark

**Affiliations:** From the ‡ Department of Microbiology, Tumor and Cell Biology, Karolinska Institutet, 171 77 Stockholm, Sweden,; the § Department of Clinical Microbiology, Karolinska University Hospital, 171 76 Stockholm, Sweden,; the ¶ Structural Biology and NMR Laboratory, Linderstrøm-Lang Center for Protein Science, Department of Biology, University of Copenhagen, Ole Maaløes Vej 5, 2200 Copenhagen N, Denmark,; the ‖ Leibniz-Institut für Molekulare Pharmakologie FMP, Robert-Rössle-Strasse 10, 13125 Berlin, Germany,; the ** Interdisciplinary Nanoscience Center (iNANO), Aarhus University, Gustav Wieds Vej 14, 8000 Aarhus C, Denmark,; the ‡‡ Aarhus Institute of Advanced Studies (AIAS), Aarhus University, Høegh-Guldbergs Gade 6B, 8000 Aarhus C, Denmark, and; the §§ Singapore Centre on Environmental Life Sciences Engineering (SCELSE) and Lee Kong Chian School of Medicine (LKC), Nanyang Technological University, Singapore 639798, Singapore

**Keywords:** microbiology, nuclear magnetic resonance (NMR), protein structure, transformation, type IV pili, Streptococcus pneumoniae, horizontal gene transfer, major pilin, pneumococci

## Abstract

Type IV pili are important virulence factors on the surface of many pathogenic bacteria and have been implicated in a wide range of diverse functions, including attachment, twitching motility, biofilm formation, and horizontal gene transfer. The respiratory pathogen *Streptococcus pneumoniae* deploys type IV pili to take up DNA during transformation. These “competence pili” are composed of the major pilin protein ComGC and exclusively assembled during bacterial competence, but their biogenesis remains unclear. Here, we report the high resolution NMR structure of N-terminal truncated ComGC revealing a highly flexible and structurally divergent type IV pilin. It consists of only three α-helical segments forming a well-defined electronegative cavity and confined electronegative and hydrophobic patches. The structure is particularly flexible between the first and second α-helix with the first helical part exhibiting slightly slower dynamics than the rest of the pilin, suggesting that the first helix is involved in forming the pilus structure core and that parts of helices two and three are primarily surface-exposed. Taken together, our results provide the first structure of a type IV pilin protein involved in the formation of competence-induced pili in Gram-positive bacteria and corroborate the remarkable structural diversity among type IV pilin proteins.

## Introduction

Type IV pili are important virulence factors on the surface of many pathogenic bacteria. These extracellular appendages can be several microns long and are involved in various functions, including adherence (1, 2), twitching motility (3, 4), biofilm formation (5, 6), and DNA uptake (7–9). Type IV pili are composed of thousands of copies of major pilin protein that are tightly packed in a helical arrangement (10, 11). Pilins are synthesized as prepilins containing a conserved N-terminal prepilin cleavage motif. Once synthesized, prepilins are processed by a membrane-bound prepilin peptidase, often called PilD, which removes the signal peptide. Based on the length of the signal peptide and the length of mature pilin, two subclasses, namely type IVa and type IVb pilins, have been distinguished (12).

A number of pilin structures are available for both subclasses mainly for Gram-negative bacteria (13). They suggest an overall conserved architecture, with each pilin having an extended N-terminal domain (α1-N and α1-C) and a C-terminal globular head domain. α1-N is primarily hydrophobic and retains the pilin subunits in the inner membrane until assembly, whereas the α1-C is tightly packed against the head domain composed of several β-strands. The α/β loop connects the N-terminal helix to the β-sheet and is important for interactions between individual pilin subunits (10). Upon pilus assembly, α1-N forms the core of the assembled pilus, and α1-C is buried in the C-terminal head domain that forms the pilus surface. Characteristic for most pilins is also a disulfide-bonded loop (D-region) in the C-terminal domain, which is essential for pilus assembly (10). Most of the structural diversity among different pilins lies in the α/β loop, and the number and topology of β-strands are in the C-terminal domain. Notably, many of the available pilin structures are lacking the highly hydrophobic N-terminal domain (α1-N) making the truncated protein more soluble and easier to purify for later structural characterization.

Type IV pili are also produced by Gram-positive bacteria, including several *Clostridium* species (14), *Ruminococcus albus* (15), and *Streptococcus* species (9, 16, 17), but many molecular and structural aspects of pilus biogenesis in Gram-positive species remain unclear. Recently, DNA uptake in *Streptococcus pneumoniae* was shown to rely on the formation of a type IV pilus that is able to directly bind to DNA (9). This transformation pilus is assembled on the surface of competent bacteria and composed of the major pilin ComGC. Pneumococcal *comGC* is encoded in the *comG* operon that also encodes a putative ATPase (ComGA), which powers pilus assembly (9), a membrane-spanning protein (ComGB), and four minor pilins (ComGD, -E, -F, and -G) whose functions remain elusive.

Herein, we characterize the pneumococcal major pilin ComGC and its ability to assemble into type IV pili. We also present the NMR structure of N-terminally truncated ComGC, which exclusively consists of α-helical segments and a variable C-terminal domain with no sequence similarity to previously characterized type IV pilin proteins.

## Results

### ComGC is the major pilin in S. pneumoniae competence-induced pili

**Figure 1.**
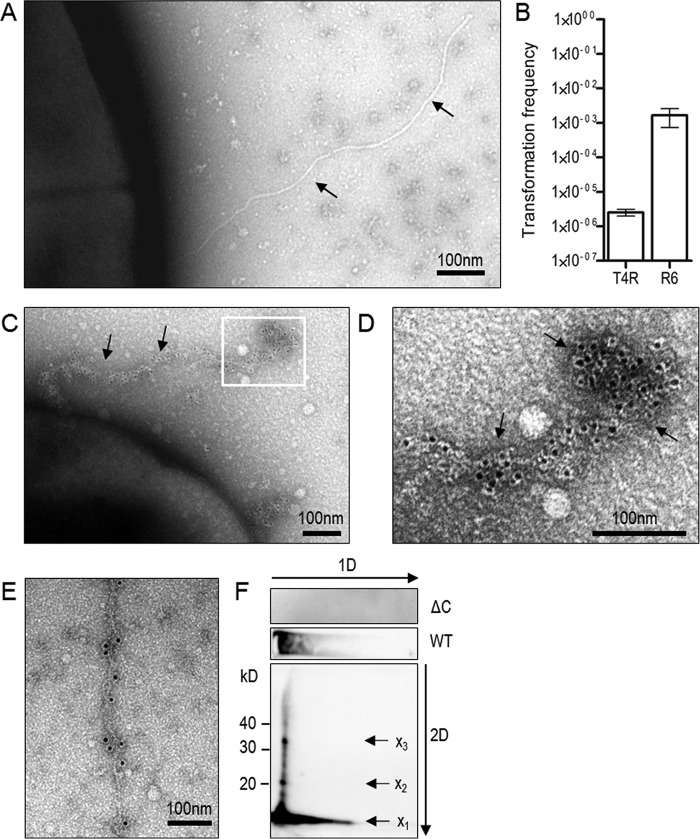
***S. pneumoniae* assembles competence type IV pili composed of ComGC.** *A,* electron micrograph of negatively stained competent *S. pneumoniae* T4RΔ*rrgA-srtD* induced with CSP. *Black arrows* indicate the pilus. *B,* transformation frequency of *S. pneumoniae* T4R and R6 strain. The *error bars* represent standard deviation (S.D.) of a minimum of three independent experiments. *C* and *D*, immunogold electron microscopy to visualize pili on competent *S. pneumoniae* R6 using primary antibody specific to ComGC and secondary antibody conjugated to 6-nm gold particles. *D,* enlargement of the immunogold-labeled pilus. *Black arrows* indicate the pilus. *E,* electron micrograph of a competence pilus in strain R6 stained with anti-ComGC antibody and protein A coupled to 10-nm gold particles. *F*, two-dimensional PAGE to assess multimerization of mature ComGC. A pilus preparation of T4 WT or ΔC strain was run on a 12% native gel (*1D,* first dimension). A piece of gel corresponding to one lane of the gel was cut and placed horizontally on top of a second SDS-PAGE (*2D*, second dimension). After migration, gels were immunoblotted with anti-ComGC antibody. *Arrows* indicate ComGC and protein multimers.

Previously it was reported that *S. pneumoniae* produces type IV pili composed of ComGC in *S. pneumoniae* strain R6 and the clinical isolates G54 and CP strains (9). To detect competence-induced pili in the *S. pneumoniae* TIGR4 (T4) background, we have used the un-encapsulated T4 strain (T4R) deficient in the *rlrA* operon (T4RΔ*rrgA-srtD*). The *rlrA* operon encodes an adhesive pneumococcal pilus that is assembled by pilus-associated sortases (18, 19). By using this mutant strain, we were able to rule out other pilus structures expressed on the bacteria. We then looked at the formation of type IV pili in T4RΔ*rrgA-srtD* cultures induced with the competence-stimulating peptide (CSP)^3^ and control cultures without CSP addition. As shown in Fig. 1*A*, a type IV pilus could be visualized by transmission electron microscopy in negatively stained *S. pneumoniae* T4RΔ*rrgA-srtD.* When we compared electron micrographs of negatively stained competent T4RΔ*rrgA-srtD* to R6, pili were less frequently observed in the T4R background, which likely provides an explanation as to why the transformation frequency is almost three orders of magnitude lower in T4R than in R6 (Fig. 1*B*).

For that reason, we decided to do immunogold labeling of ComGC in competent R6 bacteria and used primary polyclonal ComGC antibodies, raised against the purified protein or an anti-peptide antibody, and secondary antibody labeled with 6-nm gold particles. We frequently found gold particles labeling the entire type IV pilus suggesting that ComGC is the major pilin protein (Fig. 1, *C* and *D*). We also stained competent R6 bacteria with primary polyclonal ComGC antibody followed by incubation with protein A coupled to 10-nm gold particles. In this way the pilus is less frequently labeled with gold particles; however, the underlying pilus filament is clearly visible (Fig. 1*E*).

To further study pilus polymerization also in an encapsulated T4 background, we analyzed pili preparations by two-dimensional (2D) PAGE. First, pili preparations of wild-type T4 (WT) or a *comGC* knock-out mutant (ΔC) were run on a 12% native gel, which resulted in a local concentration of ComGC on the top of the gel. One lane of each sample was then cut and placed horizontally on SDS-PAGE. After migration, the gel was immunoblotted and probed with ComGC antibodies. When entering the SDS-containing gel, high-molecular-weight structures will be denatured, which is why we observe monomeric ComGC (*x*_1_); when only partially denatured, distinct ComGC building blocks (*x*_2_ and *x*_3_) can be detected (Fig. 1*F*) suggesting that ComGC also forms the pilus backbone in *S. pneumoniae* T4.

### Structural features of pneumococcal ComGC filaments

**Figure 2.**
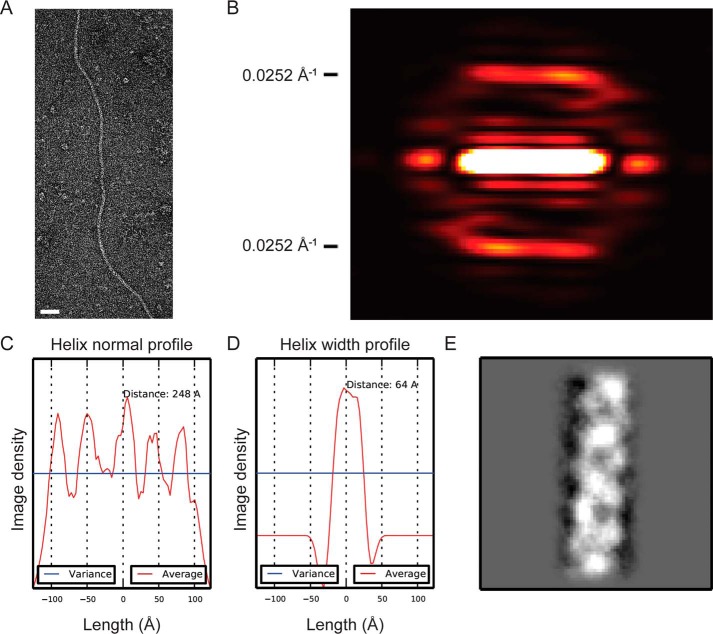
**Structural features of ComGC filaments.** *A,* representative electron micrograph of a competence pilus in strain R6 used for class averages. *Scale bar,* 50 nm. *B,* averaged power spectrum of a representative class average based on straight pilus regions. Predominant layer lines at 0.0252 Å^−1^ from the equator are visible corresponding to a helical pitch of ∼40 Å. *C,* helix normal profile along the helical axis showing a distance between peaks corresponding to the pitch derived from the power spectrum. *D,* helix width profile of class averages showing a width of 64 Å. The mean diameter of 16 class averages was 63 ± 1.6Å. *E,* representative 2D class average of the competence pilus.

To further assess the structural features of native competence pili, micrographs of uranyl acetate-stained samples of *S. pneumoniae* strain R6 were inspected. The filaments showed a pronounced degree of flexibility and only short straight regions were observed (Fig. 2*A*). A suitable amount of straight filament regions were identified by inspecting a large number of micrographs and were used for analysis of potential helical symmetry in the filaments. No helical diffraction pattern was evident from raw micrographs, but after averaging filament segments layer lines became visible in the power spectra. Based on a class average power spectrum the first prominent layer line was observed at a distance of 0.0252 Å^−1^ from the equator corresponding to a helical pitch of ∼40 Å (Fig. 2*B*). This was in accordance with a helix normal profile plot in real space showing similar distances between peaks along the helical axis (Fig. 2*C*). The average diameter of the filaments was derived from helix width profiles of 16 class averages and found to be 64 ± 1.6 Å (Fig. 2*D*). A representative class average can be seen in Fig. 2*E*.

### ComGC processing and dimerization in the membrane

**Figure 3.**
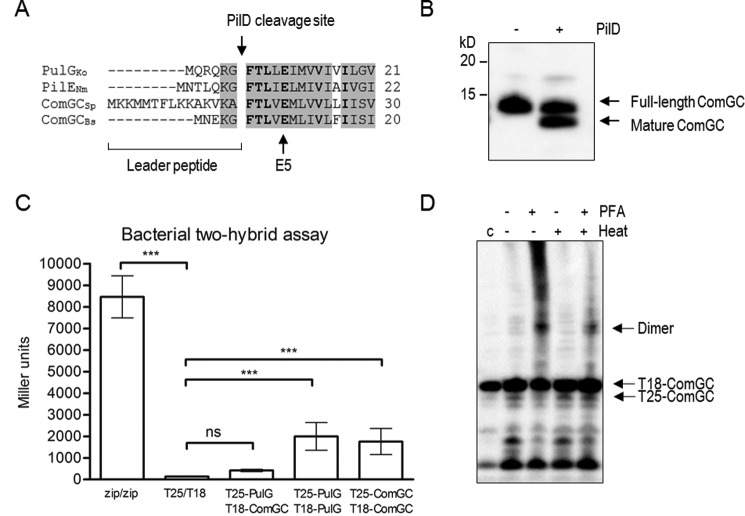
**Pneumococcal ComGC is processed by PilD and can interact with itself.** *A,* partial sequence alignment of the N-terminal domains of the major pilin proteins in *K. oxytoca* (PulG), *N. meningitidis* (PilE), *S. pneumoniae* (ComGC_Sp_), and *B. subtilis* (ComGC_Bs_). The characteristic prepilin cleavage site recognized by the prepilin peptidase PilD and the invariant Glu residues at position 5 (E5) after the cleavage site are indicated by an *arrow*. Conserved residues are *shaded,* and identical residues are shown in *bold. B, in vitro* ComGC cleavage analyzed by Western blotting with antibodies specific to ComGC. ComGC is partially processed when co-expressed with PilD in *E. coli. C,* quantification of ComGC–ComGC interaction identified by bacterial two-hybrid assay. The *graph* shows mean values of β-galactosidase activity expressed in Miller units between the indicated hybrid proteins T25-ComGC/T18-ComGC. A strain expressing Zip-T18 and Zip-T25, in which the hybrid proteins interact through a leucine zipper motif, was used as positive control. *K. oxytoca* PulG, T25-PulG/T18-PulG was included as functional positive control. *E. coli* BTH101 co-transformed with pUT18C and pKT25 empty plasmid or a strain expressing T25-PulG/T18-ComGC was used as negative control. The *error bars* represent S.D. of a minimum of three independent experiments with three different clones. A one-way analysis of variance test followed by Dunnett's post-test to compare each interaction pair to the negative control (T25/T18) was used for statistical analysis: ***, *p* < 0.001; *ns,* no significant difference. *D,* paraformaldehyde cross-linking of *E. coli* BTH101 expressing T25-ComGC/T18-ComGC. Cell extracts were analyzed by immunoblotting with anti-ComGC antibody. An untreated sample was used as expression control (indicated as *C*, *left lane*). Hybrid proteins and dimerization are indicated on the *right side* of the panel.

One characteristic of proteins belonging to the type IV pilin family is the presence of a well-conserved prepilin cleavage motif Gly-Phe-Xaa-Xaa-Xaa-Glu (20). Pneumococcal ComGC is synthesized with a 15-residue leader sequence and shares a highly conserved PilD cleavage site with other known major pilins (Fig. 3*A*). It can also be processed *in vitro* by co-expressing full-length ComGC and PilD (Fig. 3*B*).

To test whether two full-length membrane-embedded ComGC monomers can directly interact with each other, we used the bacterial adenylate cyclase two-hybrid (BACTH) system (21). Mature ComGC was fused to the C-terminal end of T25 and T18 fragments of *Bordetella pertussis* adenylate cyclase (CyaA), and *lacZ* expression was measured. Compared with the negative control (T25/T18), T25-comGC/T18-comGC showed a statistically significant increase in CyaA activity (Fig. 3*C*). Because the positive control, in which T25 and T18 are fused to the leucine zipper domain of GCN4 (21), showed much higher activity, we included another functional control protein, PulG. PulG is the major pilin protein of the type II secretion system in *Klebsiella oxyctoca* and is known to form type IV-like pili when overproduced (22). The level of CyaA activation in T25-PulG/T18-PulG was similar to T25-comGC/T18-comGC, indicating efficient dimerization of ComGC in the membrane (Fig. 3*C*). We also tested a strain expressing T25-PulG/T18-ComGC, which showed very low CyaA activity similar to the negative control (T25/T18), suggesting that these two functional major pilins cannot interact in the membrane. To validate our interaction between two ComGC monomers, we also performed chemical cross-linking of *Escherichia coli* BTH101 expressing T25-comGC/T18-comGC and were able to detect ComGC dimerization by immunoblotting with ComGC antiserum (Fig. 3*D*).

### Structure of soluble ComGC

**Figure 4.**
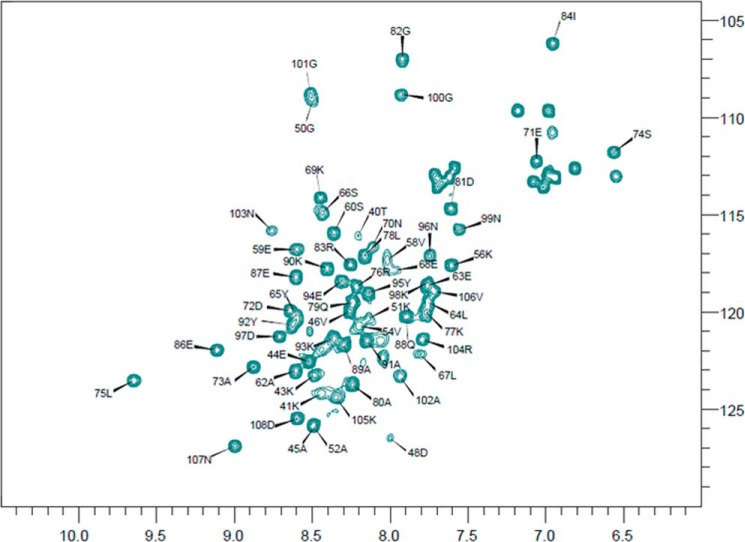
**Assigned ^1^H-^15^N HSQC of ComGC recorded at 283K.** The HSQC spectrum displays signals from all the backbone and side chain N-H correlations. The ^1^H chemical shift is plotted along the *x* axis and the ^15^N chemical shifts along the *y* axis. The chemical shifts report on the local chemical environment, and thus very small changes in structure will cause changes in chemical shifts. We observed 65 well defined amide peaks in the ComGC HSQC of which we were able to assign 61 residue-specific resonances (*black arrows*). Unassigned resonances in the *upper right corner* are side-chain N-H correlations. Importantly, we do not see any clear indication of more than one conformational state as this would give rise to more chemical shifts in the spectrum.

**Figure 5.**
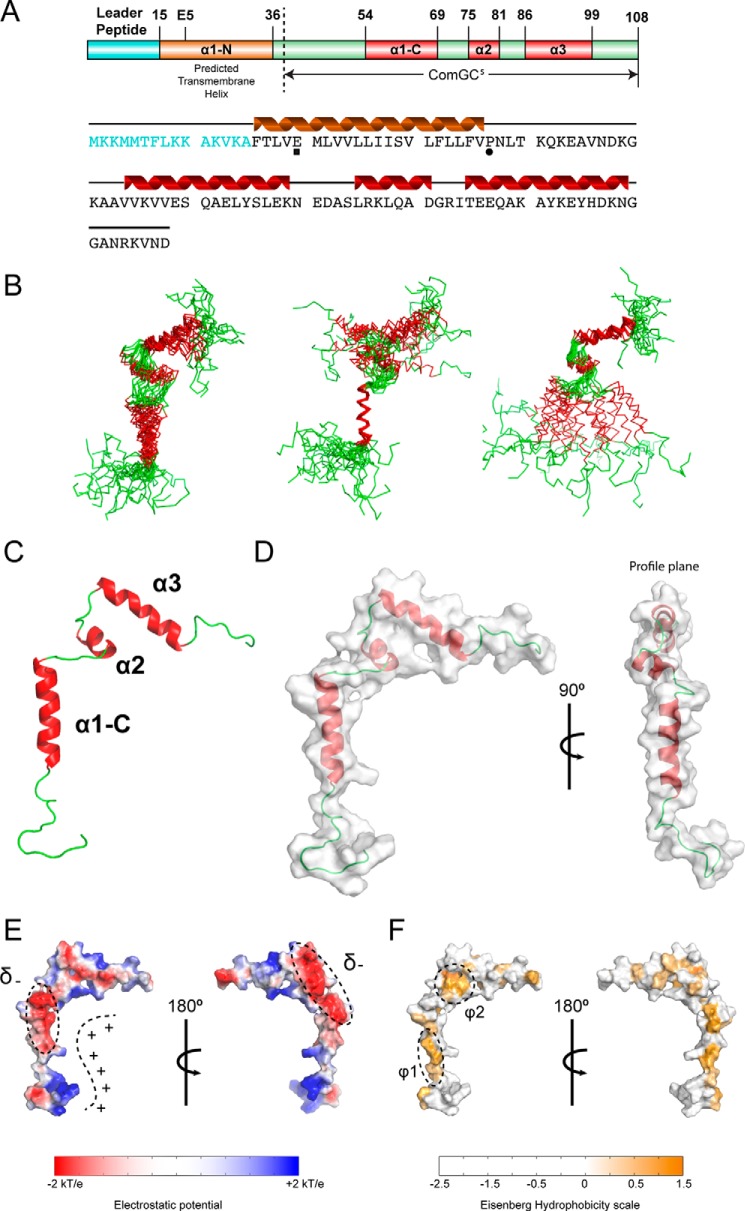
**NMR structure of soluble ComGC is composed of three flexible helical segments.** *A,* schematic and secondary structure overview of the full-length ComGC protein. The leader peptide is colored in *cyan*; the membrane-spanning helix is in *brown*; soluble helices are in *red,* and flexible regions are in *green*. The ComGC^s^ construct used for structural determination by NMR is indicated. In the protein sequence Glu-5 is marked by ■ and the helix-breaking residue Pro-22 by ●. *B,* alignment of the 20 lowest energy structures returned from the structure calculations. Ensemble alignments for all three helices (*left*), α1-C only (*middle*), and α2-α3 only (*right*) are shown. The structure is particularly flexible between α1-C and α2-α3, which is illustrated by alignment of the α1-C only and α2 and α3. Root mean square deviation values are listed in supplemental Table S1. *C,* schematic of the ComGC^s^ NMR structure showing the three helical segments as well as the flexible regions. *D,* calculated solvent-accessible surface of ComGC^s^. *E,* APBS calculated electrostatics from ±2 *kT/e* display well defined electropositive (+) and electronegative (δ^−^) solvent-accessible patches. *F,* solvent-accessible hydrophobic patches φ1 and φ2 colored using the Eisenberg hydrophobicity scale ranging from −2.5 to 1.5.

ComGC has very little sequence similarity to other pilins of which the three-dimensional structure has been solved. Specifically, ComGC has only few hydrophobic amino acid residues in the C terminus, which in other pilins form the β-strand-rich head domain. Indeed, secondary structure predictions using Jpred4 (23) and Agadir (24) show that the ComGC sequence has several segments with high α-helix propensity and no β-strand propensity (supplemental Fig. S1). This suggests that the three-dimensional structure of pneumococcal ComGC may differ significantly from known structures of type IV pilins. To determine the structure of ComGC, we prepared a truncated construct lacking the predicted N-terminal transmembrane helical domain (ComGC^s^, see Fig. 5*A*). It was previously shown for the major pilin PAK in *Pseudomonas aeruginosa* that deletion of α1-N does not perturb the structural fold, with full-length and truncated protein essentially being identical (25). To determine the ComGC^s^ structure, we used NMR spectroscopy. ComGC^s^ provided well-resolved spectra and remained largely homogeneous and stable at 10 °C (Fig. 4), which enabled us to solve the atomic resolution structure of ComGC^s^ in solution (Fig. 5*B*). A summary of the structural statistics and constraints is provided in Table 1. ComGC^s^ consists of three flexible helical segments: α1-C, involving residues 54–69; a shorter α2 helix involving residues 75–81; and finally, a C-terminal α3 spanning residues 86–99. The first 14 N-terminal and 10 C-terminal residues remain unfolded in our in-solution structure, and only few inter-residual NOEs are observed in this part of the molecule (supplemental Fig. S2). This observation is in good agreement with the secondary chemical shifts (supplemental Fig. S1*A*). α1-C seems to be only loosely attached to α2 and α3, and a general lack of long-range distance restraints between these two “domains” indicates that the structure is less constrained and rather flexible in this hinge. The relative orientation of the helices was restrained by measuring residual dipolar couplings (Fig. 5, *B* and *C*). The overall tertiary fold appears almost two-dimensional being ∼60 Å tall in the vertical plane, ∼50 Å wide in the horizontal plane, but only ∼10 Å broad in the profile plane (Fig. 5*D*), which essentially caused all three helices to be mostly solvent-exposed, providing a very large solvent-accessible surface (∼8000 Å^2^). Visualizing the electrostatic potential of the solvent-accessible surface revealed a well-defined electropositive cavity formed between the helices, and two highly electronegative areas (denoted δ^−^) in the top of α1-C and the opposite side of α2 (Fig. 5*E*). Similarly, visualizing the hydrophobicity of the surface also showed two well-defined patches. The first hydrophobic patch (φ1) is situated at the back of α1-C, opposite to the electropositive patch (φ1) and the other (φ2) in α2 (Fig. 5*F*).

**Table 1:** **NMR structural constraints and structure statistics for ComGC^s^** None of the structures exhibit distance violations of >0.3 Å or dihedral angle violations >4°.

**Restraints**	
NOE-based restraints	
Intraresidual (|*i* − *j*| = 0)	177
Sequential (|i − j| = 1)	127
Medium range (2 ≤|*i* − *j*| ≤ 4)	63
Long (|*i* − *j*| > 5)	19
Total	386
Hydrogen bond restraints	18
Dihedral angle restraints	106
Residual dipolar couplings	41
Magnitude (*D_a_*)	−15
Rhombicity (*R*)	0.362

**Restraints statistics**	
Mean r.m.s.d from experimental restraints	
NOE-based distances, Å	0.022 ± 0.007
Dihedrals, °°	0.415 ± 0.009
RDC, *Q*-factor	0.078 ± 0.006
Structure statistics*^a^*	
Most favored regions	98.1%
Allowed regions	1.9%
Generously allowed regions	0.0%
Disallowed regions	0.0%
Coordinate precision r.m.s.d, Å*^b^*	
Backbone heavy atoms (N, C^α^, and C′)	3.48 ± 0.76
Heavy atoms	4.40 ± 0.86
*H1s only* (*16–30*)	
Backbone heavy atoms (N, C^α^, and C′)	0.93 ± 0.32
Heavy atoms	1.94 ± 0.42
*H2 and H3* (*36–60*)	
Backbone heavy atoms (N, C^α^, and C′)	2.41 ± 1.16
Heavy atoms	3.49 ± 1.40

*^a^* PROCHECK, structured regions 16–30, 36–42, and 47–60 are shown.

*^b^* Residues 16–30, 36–42, and 47–60 are shown. Large root mean square deviations can be assigned to the flexible hinge between H1 and H2.

### ComGC^s^ dynamics on different time scales might be important for pilus assembly

**Figure 6.**
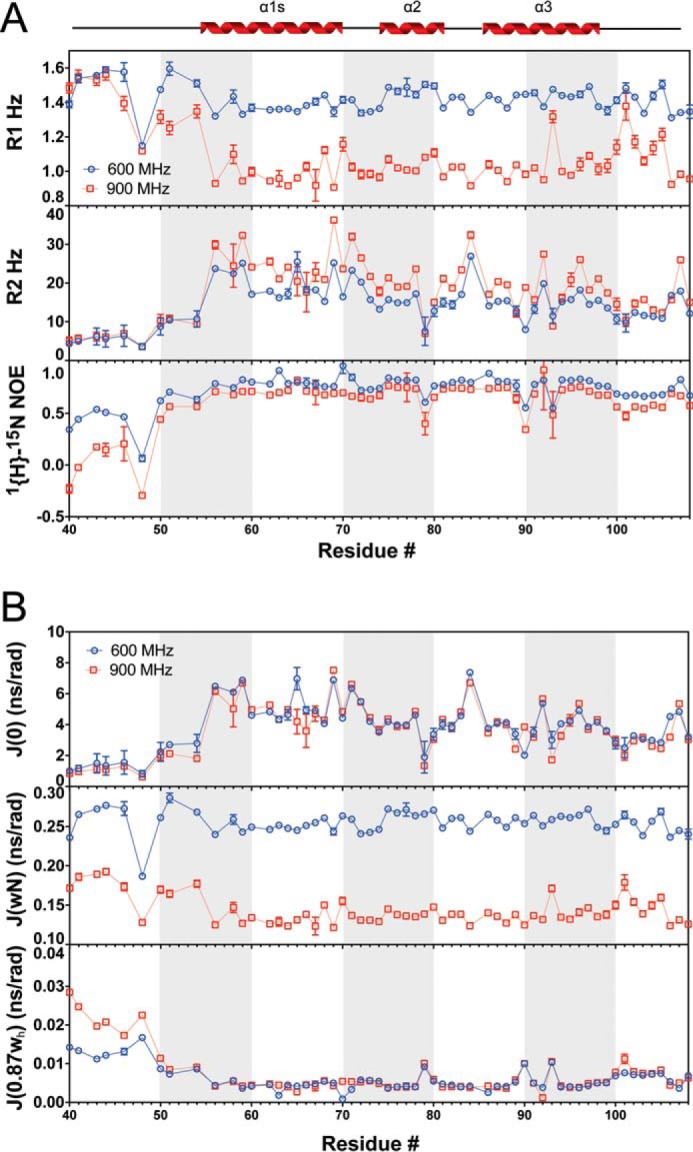
**ComGC^s^ is dynamic on several different time scales.** *A,* fitted ^15^N *R*_1_ rates (*top*), *R*_2_ rates (*middle*), and hetNOEs (*bottom*), at two different field strengths, 600 and 900 MHz, with respect to protons. The secondary structure of ComGC^s^ is shown above. *B,* reduced spectral density functions *J*(0) (*top*), *J*(ωN) (*middle*), and *J*(0.87ωh) (*bottom*) calculated from the backbone relaxation data.

The structural flexibility hypothesized above led us to study the dynamics in greater detail, and we therefore measured the longitudinal (*R*_1_) and the transverse (*R*_2_) ^15^N relaxation rates in both (relative to the external magnetic field) as well as heteronuclear ^1^H-^15^N-NOEs (hetNOEs), at two different field strengths (Fig. 6*A*). As expected, and in support of the ComGC^s^ structure, the unstructured region 40–52 displayed generally longer *R*_1_ rates (>1.5 s^−1^), short *R*_2_ rates (<10 s^−1^), and relatively lower hetNOE values (<0.5), compared with the structured regions. Unlike *R*_2_, *R*_1_ rates are strongly field-dependent, and therefore the relative *R*_1_ difference is mostly visible at the higher field strength. The *R*_2_ rates are generally high (20.3 s^−1^ on average for residues 56–108) in all of the structured regions as would be expected. However, several residues in α1-C exhibit high *R*_2_ rates, which could suggest conformational exchange with one or more additional states (Fig. 6*B*). To gain insight into site-specific internal motion, we used the measured *R*_1_, *R*_2_, and hetNOE values to calculate the reduced spectral density functions, *J*(0), *J*(ω_N_) and *J*(0.87ω_H_), reporting on dynamics on three different time scales (Fig. 6*B*). *J*(0) represents protein mobility in the nano-second time scale, thus low *J*(0) values normally indicates higher flexibility as observed for residues 40–52, showing also higher internal motion in the *J*(ω_h_) pico-second time scale supporting that this region remains largely unstructured in solution. The more structured regions, and especially the flexible hinges between the helical segments, displayed much higher *J*(0) values indicating that these regions have mobility on the nano-second time scale. Interestingly, Asp-48 just before α1-C appeared highly dynamic from the hetNOE experiment; however, on a time scale (lower nano-second) different from all other residues. Also, the hydrophobic residues Leu-78 and the stretch from Ala-89 to Lys-93, located in the interface between α2 and α3 showed dynamics on a faster time scale than observed for other nearby residues.

### Sequence variation in ComGC

**Figure 7.**
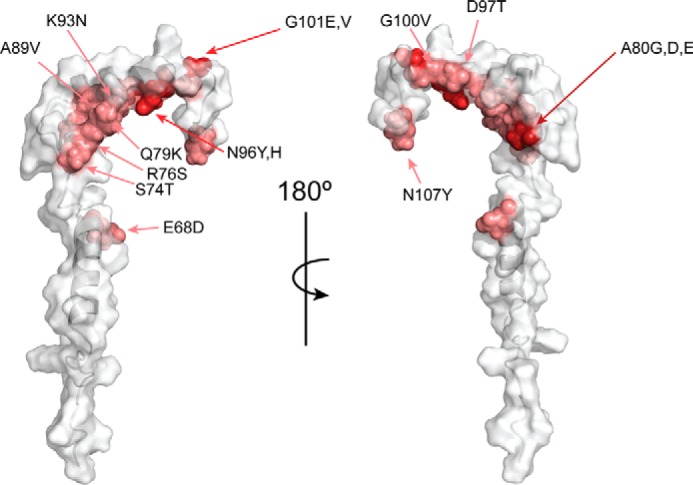
**Polymorphisms in ComGC.** Model of soluble ComGC. Polymorphisms are plotted on the ComGC structure. Variable residues are shown in shades of *red* depending on their susceptibility to undergo mutations.

A total of 14 polymorphic sites differing in the number of variations from the reference sequence TIGR4 were identified in 23 publicly available *S. pneumoniae* genomes suggesting that ComGC is well conserved. A phylogenetic tree of strains clustered according to their ComGC sequence and the corresponding multiple sequence alignment are shown in supplemental Fig. S3. The most divergent strains exhibit a sequence identity of 91%. All strains can be grouped into two main clusters. The strains belonging to cluster 1 are identical with exception of strain NT11058 that carries one additional variation (N107Y) than the other strains present in this group. The second cluster, containing our reference sequence, is more diverse and can be sub-grouped into five sub-clusters. The virulent strain D39 and the avirulent, un-encapsulated laboratory strain R6, a derivative of D39, are closely related to TIGR4 with only one sequence variation (N96H). The majority of polymorphisms are localized to the interface between helices α2 and α3 and only 1 out of 14 is localized to the αC-1 helix (Fig. 7). This suggests that the hypothesized hydrophobic pilin–pilin interface involving the transmembrane α1-N and α1-C helical domains has been largely conserved throughout evolution and that the α2-α3 head group has undergone significantly larger changes primarily affecting the exposed electrostatic regions depicted in Fig. 5*E*.

## Discussion

The pneumococcal competence pilus was first visualized recently (9). It is morphologically similar to other type IV pili described displaying filament diameters between 6–9 nm (11). Competence pili in *S. pneumoniae* have a mean diameter of 64 Å, which is comparable with the 60 Å diameter of type IV pili in *Neisseria gonorrhoeae* (26). They are helical assemblies, and the observed pitch of ∼40 Å is somewhat larger than the 37 Å pitch of *N. gonorrhoeae* pili, suggesting different assembly and stabilization strategies in ComGC competence pili. In comparison, the type IV pilus in *Thermus thermophilus*, ∼3 nm in diameter, shows a helical pitch of 49 Å forming a less compact pilus than type IV pili in *N. gonorrhoeae* (27). The observed differences in pilus diameter and helical pitch are likely explained by structural features of the major pilin subunit, which can vary considerably in sequence and size among bacteria expressing type IV pili.

Pneumococcal ComGC shares many features of canonical type IV pilins. Full-length ComGC has a well-defined conserved prepilin cleavage motif, an invariant Glu residue at position 5 after the cleavage site, and it is processed by PilD. The structure of soluble ComGC, provided here, is the first example of a type IV pilin protein involved in the formation of competence-induced pili in Gram-positive bacteria and reveals new structural features. Similar to previously described type IV pilins, ComGC has a predicted extended N-terminal α-helix but differs otherwise significantly as follows: 1) ComGC is exclusively α-helical; 2) the head group is much smaller; 3) the α1-C helix is separated from the transmembrane helix by a flexible linker that is largely unfolded in solution; and 4) ComGC contains no cysteines. Overall, ComGC is shorter than other type IV pilins and highly dynamic in solution, which may be an important feature for pilus assembly and function.

An important question raised by this structure regards the stabilization of ComGC. Most type IV pilins in Gram-negative bacteria and the major pilin ComGC in *Bacillus subtilis* have two cysteine residues in the C-terminal part of the protein that are important for protein stability and polymerization (13, 28), but there is no disulfide bond to stabilize ComGC. The major pilin, PilA1, in the Gram-positive bacterium *Clostridium difficile* also lacks cysteines (29). However, it is structurally much more compact in its C terminus than pneumococcal ComGC. In ComGC only two α-helices (α2 and α3) are forming the head domain, and the absence of other stabilizing structural elements might explain the observed flexibility in this region. In fact, PilA of *Geobacter sulfurreducens* (only 66 amino acids) is essentially lacking any globular head domain, and the NMR structure also showed a highly dynamic C-terminal region (30).

The assembly of pilin monomers into a model of the fully formed pilus has primarily been based on negative staining and electron cryo-micrographs, where individual monomers are fixed in a favorable multimeric organization and fitted into the obtained electron density (26, 31). These structural models serve as important frameworks for understanding pilus dimensions, appearance, and surface, but as a consequence of the low structural similarity of ComGC, primarily in the head group, we were not able to reliably predict ComGC assembly. Our data suggest that soluble monomeric ComGC will not adopt secondary or tertiary folds similar to other type IV pilins, but we cannot rule out that additional conformational states (*i.e.* helical rearrangements) will be favored during assembly or in the mature pilus structure.

The structure of ComGC itself provides initial information on the assembly and function of competence pili in *S. pneumoniae*. The electrostatic potential as well as the hydrophobicity of the accessible surface in ComGC reveals highly defined patches, which might restrict or guide pilus formation. Based on the hydrophobicity profile, we propose that α1-N and α1-C are involved in forming the core of the pilus structure, with parts of α2 and α3 being primarily surface-exposed. The residues Leu-78, Ile-84, and Tyr-92, involved in the hydrophobic patch, φ2, formed between α2 and α3 seem functionally distinct and may contribute to pilin flexibility during pilus assembly. Additionally, they could provide better resistance to shear forces in the environment by increasing the flexibility of the assembled pilus. It is also notable that the proposed α1-N and α1-C helices in ComGC seem to be separated by a larger stretch of residues, including the helix-breaking residue Pro-22, with no or less helical propensity. Interestingly, cryo-electron microscopy reconstruction of the *Neisseria meningitidis* type IV pilus recently revealed a similar non-helical portion in α1-N, between the residues Gly-14 and Pro-22, of the major pilin pilE (31). This stretch was proposed to function as a spring providing the filament additional flexibility in response to external forces, and it may have a similar purpose in pneumococcal competence pili.

Type IV pili have a conserved role during the process of transformation, and pilus-deficient strains of naturally transformable species have reduced DNA uptake potential (32–34). Interestingly, *Neisseria* species bind DNA, in a sequence-specific manner, through the minor pilin ComP exposed on the type IV pilus surface (35, 36). In many other competent bacteria, the exact mechanisms that govern pilus-DNA interactions remain elusive. It is generally believed that DNA binding is a function of the intact pilus through solvent-exposed surface residues that mediate interactions with the DNA backbone. Laurenceau *et al.* (9) have previously shown direct DNA binding to the pneumococcal pilus, but monomeric ComGC was unable to bind DNA (37) suggesting that elements in the competence pilus quaternary structure are required for DNA interactions. Visualizing the electrostatic potential of the solvent-accessible surface in ComGC revealed a well-defined electropositive cavity formed between the helices. Along with the flexible N-terminal part, this region displays several solvent-exposed Lys and Arg residues, which are residues that have been found to mediate DNA backbone interactions in other DNA-binding proteins (38). Once a competence pilus model is available, it will be interesting to explore whether and how this electropositive cavity contributes to DNA binding.

In conclusion, the structure of pneumococcal ComGC represents a unique member in the growing family of type IV pilins, and it provides initial structural insights into understanding how competence pili assemble and how DNA is taken up in natural transformation of *S. pneumoniae*.

## Experimental procedures

### Bacterial strains and growth conditions

All *S. pneumoniae* strains used in this study are described in supplemental Table S1. Bacteria were grown on blood agar plates at 37 °C and 5% CO_2_ overnight (O/N). For competence induction, plate-grown bacteria were used to inoculate C+Y medium, pH 7.9–8.0, at *A*_620_ = 0.05 and grown without agitation at 37 °C until *A*_620_ = 0.15. Competence was induced by addition of competence stimulating peptide (CSP-1 or 2 dependent on the strain used) at a final concentration of 100 ng/ml for 20 min, if not specified otherwise.

### Transmission electron microscopy and immunogold labeling to visualize competence pili

*S. pneumoniae* R6 or T4RΔ*rrgA-srtD*, an unencapsulated strain lacking pili encoded by the *rlrA* islet, were grown at 37 °C in C+Y medium until *A*_620_ = 0.15 when competence was induced as described above. Twenty minutes post-induction the cells were centrifuged for 15 min at 5000 × *g*, 4 °C. The pellet was resuspended in 80 μl of phosphate-buffered saline (PBS). Drops of 10 μl were placed for 1 min on glow-discharged carbon-coated copper grids (Oxford Instruments, UK) for negative staining or carbon-coated gold grids (Aurion, Germany) for immunogold labeling. Negative staining was performed with 2% uranyl acetate in water. For immunogold labeling anti-ComGC antiserum, raised against a synthetic peptide corresponding to residues 95–108 of ComGC, was used. Grids were fixed with 10 μl of 0.2% glutaraldehyde for 2 min, and the reaction was stopped with 10 μl of 1% glycine for 15 min. The grids were then washed three times with PBS, 1% BSA, incubated with ComGC antibodies (1:100) for 1 h, washed three times with PBS, and incubated with secondary goat anti-rabbit antibody conjugated to 6-nm gold particles or protein A coupled to 10-nm gold particles diluted 1:250 for 45 min. Finally, the grids were washed six times with PBS and twice with distilled water before negatively staining with 2% uranyl acetate. Specimens were examined in a Tecnai 12 Spirit Bio TWIN transmission electron microscope (FEI Company, Eindhoven, Netherlands) operated at 100 kV. Digital images were recorded using a Veleta camera (Olympus Soft Imaging Solutions, GmbH, Münster, Germany).

### Analysis of competence pili

Negative stain grids were prepared as described under immunogold labeling. Micrographs were collected manually at 120 kV using a Tecnai G2 Spirit TWIN electron microscope with a defocus value of 0.5–2.0 μm. Images were collected using a Tietz TemCam-F416 CMOS camera at a nominal magnification of ×67,000 and a pixel size of 1.57 Å employing the EM-Menu software (TVIPS GmbH). Data processing was done using the SPRING suite employing CTFFIND, CTFTILT, EMAN2, and SPARX (39–43). Straight regions of the pilus filaments were extracted, segmented, and averaged to determine outer dimensions and helical parameters. Averaged intensity width profiles were plotted, and the outer diameter was taken as the distance between the two outer minima in the intensity profile. Power spectra were calculated from the averages, and the most prominent layer lines were identified. The pitch was determined in real space from intensity normal profiles along the helical axis as well as from Fourier space based on layer line positions.

### Preparation of pili, two-dimensional PAGE, and immunoblotting to assess ComGC assembly

Competence pili preparations were obtained from *S. pneumoniae* grown in 500 ml of C+Y medium, and competence was induced as described above. Bacteria were pelleted at 4 °C by centrifugation for 15 min at 6000 × *g*. The supernatant containing detached/broken pili was filtered and pelleted by ultracentrifugation at 100,000 × *g* at 4 °C for 1 h. Pellets were resuspended in 100 μl of PBS. Multimerization of mature ComGC was assessed by two-dimensional PAGE, native gel (first dimension) and SDS-PAGE (second dimension). In brief, pili preparations of competent T4 WT and T4ΔcomGC were run on a 12% native gel. Then, one entire lane of each sample was cut and placed perpendicular on top of a second gel. After migration, electroblotting (Bio-Rad, Trans-Blot® Turbo^TM^ Midi PVDF Transfer Packs) and immunodetection with ComGC antibody were performed. Rabbit polyclonal ComGC antibody has been previously described (37). HRP-conjugated goat anti-rabbit antibody (GE Healthcare) and Amersham Biosciences ECL Prime Western blotting detection reagent (GE Healthcare) were used to visualize the blots.

### Transformation frequency assay

Genomic DNA of *S. pneumoniae* carrying a streptomycin resistance mutation in the *rpsL* gene (44) was used to transform competent bacteria. In brief, *S. pneumoniae* was grown in C+Y medium at 37 °C until *A*_620_ = 0.15. Bacteria were then incubated at 30 °C for 15 min before CSP was added. After 15 min, 1 μg/ml DNA was added. Bacteria were then incubated for 30 min at 30 °C and another 60 min at 37 °C before plating in the presence and absence of streptomycin at 100 μg/ml final concentration. Blood plates were incubated O/N at 37 °C and 5% CO_2_ before being counted.

### In vitro ComGC processing

The plasmids pJWV25-*PilD* and pACYCDuet-1-*flcomGC* were constructed as follows. Full-length *pilD* and full-length *comGC* were amplified from *S. pneumoniae* TIGR4 genomic DNA using Phusion Flash High-Fidelity PCR Master Mix (Thermo Fisher Scientific) and suitable primers (supplemental Table S2). PCR products were digested with NotI (*pilD*) or NdeI and Xho (*comGC*) and subcloned into pJWV25 and pACYCDuet-1, respectively. The correct insertion was confirmed by PCR and sequencing (Eurofins MWG Operon). The resulting plasmids pJWV25-*PilD* and pACYCDuet-1-*flcomGC* (supplemental Table S3) were then co-transformed into competent T7 express *Escherichia coli* (New England Biolabs). Bacteria were grown in LB, pH 7.5, supplemented with 100 μg/ml ampicillin and 50 μg/ml chloramphenicol at 37 °C until *A*_600_ = 0.5, and induced with 1 mm isopropyl β-d-thiogalactopyranoside for 3 h. Bacteria were spun down, and 1× sample buffer was added to the pellet. Samples were incubated at 100 °C for 5 min before analysis by SDS-PAGE and immunoblotting with ComGC antibody as described above.

### BACTH

All plasmids used for BACTH are listed in supplemental Table S3. The gene encoding mature ComGC and mature PulG were PCR-amplified using suitable primers (supplemental Table S2) and cloned into pUT18C and pKT25. *E. coli* Top10 (Invitrogen) was used for all clonings. *E. coli* BTH101 (Euromedex) was co-transformed with respective BACTH plasmids (supplemental Table S3) and used for BACTH assay. The efficiency of the functional complementation between the recombinant plasmids encoding fusions to T18 (pUT18C) and T25 (pKT25) was quantified by measuring β-galactosidase activity in liquid culture as described previously with some modifications (21). Co-transformed BTH101 strains were grown in 5 ml of LB medium, supplemented with 100 μg/ml ampicillin, 50 μg/ml kanamycin, and 0.5 mm isopropyl β-d-thiogalactopyranoside, O/N at 30 °C. Three individual clones of each co-transformation were tested, and at least three independent cultures were performed. Subsequently, cultures were incubated for 20 min on ice and pelleted by centrifugation for 10 min at 4 °C. Next, cells were resuspended in the same volume of 1× Z buffer (90 mm Na_2_HPO_4_·2H_2_O, 40 mm NaH_2_PO_4_·H_2_O, 6 mm NaOH, 10 mm MgSO_4_·7H_2_O, 50 mm β-mercaptoethanol) and diluted until a final *A*_600 nm_ = 0.3. Then, 1 ml of bacterial suspension was permeabilized by adding 100 μl of chloroform and 50 μl of 0.1% SDS. Tubes were then vortexed and incubated at 28 °C for 10 min before the enzymatic reaction was started by adding 200 μl of 0.4% *o-*nitrophenyl-β-d-galactopyranoside in phosphate buffer (90 mm Na_2_HPO_4_·2H_2_O, 40 mm NaH_2_PO_4_·H_2_O). The reaction was stopped by the addition of 500 μl of 1 m Na_2_CO_3_ when the samples became noticeably yellow, and the time of incubation with the substrate was recorded. The reaction mixtures were centrifuged for 5 min, and the supernatants were transferred into a cuvette. Then, the absorbance was recorded at 420 and 550 nm for each sample. The β-galactosidase activity was expressed in Miller units by using the following formula: 1000 × (*A*_420 nm_ − 1.75 × *A*_550 nm_)/(incubation time (minutes) × volume (1 ml) × *A*_600 nm_).

### Chemical cross-linking

*In vitro* cross-linking experiments were essentially performed as described previously (46). In brief, exponentially grown bacteria were pelleted by centrifugation, washed with 10 mm sodium phosphate buffer, pH 6.8, and incubated with 1% paraformaldehyde (Sigma) in 10 mm sodium phosphate buffer, pH 6.8, for 30 min. Cross-linking was stopped by addition of 3 m Tris, pH 8.8 (final concentration 300 mm Tris). Bacteria were washed, and pellets were resuspended in 1× NuPAGE sample buffer (Thermo Fisher Scientific) without reducing agent. Each sample was split into two tubes. One tube was kept at room temperature, and one tube was heated at 96 °C for 15 min before further analysis by SDS-PAGE and Western blotting with ComGC antibody.

### Expression and purification of labeled ComGC for NMR

The DNA sequence of ComGC lacking the signal peptide and codons for the N-terminal hydrophobic domain (ComGCΔ1–39) was cloned downstream of the His_6_ tag sequence into pet28a vector (Novagen). Constructs were confirmed by sequencing and transformed into *E. coli* Rosetta (DE3). Cells were grown O/N with shaking at 37 °C in M9 minimal media supplemented with [^13^C]glucose and [^15^N]ammonium sulfate containing 50 μg/ml kanamycin. The O/N culture was then diluted into fresh medium; cells were grown to *A*_620_ = 0.5 at 37 °C and induced with 1 mm isopropyl β-d-1 thiogalactopyranoside for 3.5 h. Cells were harvested by centrifugation at 7000 × *g* for 20 min at 4 °C, and pellets were stored at −20 °C. For affinity purification of ComGC, cell pellets were resuspended in buffer containing 50 mm Tris, 50 mm NaCl, pH 7.5, and protease inhibitor (Roche Applied Science) and lysed in a Stansted cell disrupter. Unbroken cells were pelleted by centrifugation at 35,000 × *g,* and supernatants were incubated with nickel-nitrilotriacetic acid (Ni-NTA, Qiagen)-agarose with rotation at 4 °C O/N. After washing the resin, protein was eluted with buffer containing 50 mm Tris, 50 mm NaCl, and 250 mm imidazole, pH 7.5. Imidazole was removed using PD-10 desalting columns (GE Healthcare). The N-terminal His_6_ tag was cleaved with thrombin (Sigma) for 2 h at room temperature and removed by incubation with Ni-NTA resin. ComGC was further purified by size-exclusion chromatography on a Superdex 75 gel filtration column.

### Nuclear magnetic resonance (NMR)

#### 

##### NMR data collection

The NMR samples contained ComGC at a concentration of 0.9 mm in a buffer containing 50 mm Tris, 50 mm NaCl, pH 7.5, and had a volume of 800 μl. Experiments were performed in 5-mm tubes at a temperature of 283 K on Bruker AVIII 600 spectrometer operating at 600 MHz (^1^H frequency). They were equipped with either a TXI cryoprobe or a TCI cryoprobe both equipped with a self-shielding z-gradient, and a Bruker AVII 900 spectrometer operating at 900 MHz (^1^H frequency), also at 283 K, and using a TCI cryoprobe equipped with a self-shielding z-gradient. Residual dipolar coupling (RDC) and HCCH-TOCSY spectra were recorded on an 800 MHz Agilent DD2 spectrometer with a room temperature probe.

##### Sequential assignment

ComGC backbone chemical shifts were sequentially assigned using ^1^H,^15^N-HSQC, ^1^H,^13^C-HSQC CBCANH, CBCA(CO)NH, HNCA, HN(CO)CA, HNCO, HN(CA)CO, HBHA(CO)NH CC(CO)NH, and HCC(CO)NH spectra. Side-chain chemical shifts were assigned by using a HCCH-TOCSY spectrum recorded with a mixing time of 20 ms, and ^15^N-TOCSY-HSQC and ^13^C-TOCSY-HSQC spectra recorded with mixing times of 80 ms. Chemical shifts are deposited in the BMRB with ID 34112.

##### Structural determination

Distance restraints were obtained from ^15^N NOESY-HSQC and ^13^C NOESY-HSQC experiments recorded using mixing times of 150 and 80 ms, respectively. Residual dipolar couplings were obtained from a sample aligned in PEG (C_12_E_5_)/hexanol (Sigma: 76437/H13303) liquid crystal medium with a final PEG concentration of 4%. The couplings were measured in in-phase/anti-phase ^1^H,^15^N HSQC spectra (47–49). Backbone dihedral angle restraints were calculated using DANGLE (50). Automated NOE assignment was performed using Cyana (51), and XPLOR-NIH (52) was subsequently used for including the RDC restraints and refining the structure. In total, 200 structures were calculated from which the 20 lowest energy structures were selected. Structures were aligned using Theseus (53) and visualized in PyMOL (DeLano Scientific). Electrostatics were calculated using APBS (54). Ramachandran plot statistics for the structural ensemble were calculated with PROCHECK (55). The coordinates are deposited in the Protein Data Bank with code 5NCA.

##### Dynamics

*R*_1_ and *R*_2_ relaxation rates were measured in using the 2D ^1^H,^15^N HSQC-based pulse sequences by Farrow *et al.* (56) using delays of 0.080*, 0.240, 0.400*, 0.640, 0.880*, 1.280, and 1.600 s for *R*_1_ experiments and 0.016*, 0.032, 0.048, 0.08*,0.112, 0.144*, and 0.2 s for *R*_2_ (starred values are recorded in duplicates). Heteronuclear ^1^H-^15^N NOE values were determined from peak ratios between saturated/steady-state and a reference spectrum (*I*_ss_/*I*_ref_) (57). Reduced spectral densities are calculated using Equations 1–4,

(Eq. 1) $$J\left(0\right)=\frac{1}{3d^{2}+4c^{2}}\left(6R_{2}-R_{1}\left(3-\frac{18}{5}\left(\frac{\gamma_{\text{N}}}{\gamma_{\text{H}}}\right)\left(\text{NOE}\right)-1\right)\right)$$

(Eq. 2) $$J\left(\omega_{\text{N}}\right)=\frac{4}{3d^{2}+4c^{2}}\left(R_{1}\left(1-\frac{7}{5}\left(\frac{\gamma_{\text{N}}}{\gamma_{\text{H}}}\right)\left(\text{NOE}\right)-1\right)\right)$$

(Eq. 3) $$J\left(0.87\omega_{h}\right)=\frac{4}{5d^{2}}\left(R_{1}\left(\frac{\gamma_{\text{N}}}{\gamma_{\text{H}}}\right)\left(\text{NOE}-\text{1}\right)\right)$$

(Eq. 4) $$d=\left(\frac{\mu_{0}h\gamma_{\text{N}}\gamma_{\text{H}}}{8\pi^{2}}\right)\frac{1}{r{\text{NH}}^{3}},c=\frac{\omega_{\text{N}}\Delta \sigma }{\sqrt{3}}$$

where *R*_1_ and *R*_2_ and NOE are the fitted relaxation rates or intensity ratio; γ_N_ and γ_H_ are the gyromagnetic ratios of N and H; μ_0_ is the permeability of free space; *h* is Planck's constant; *r*_NH_ is the bond length vector of the amide NH bond (set to 1.02 Å); ω_N_ is the larmor frequency of N, and Δσ is the chemical shift anisotropy of N (set to −172 ppm) (45).

### Statistical analysis

Data were statistically analyzed as indicated in the figure legends, using GraphPad Prism 5.04. If not stated otherwise, asterisks in the figures indicate groups of statistically different means (***, *p* < 0.001), determined by one-way analysis of variance with subsequent Dunnett's post hoc test.

## Author contributions

S. M., U. A., and B. H. N. designed the study, and S. M., M. S. A., and V. O. performed the experiments. S. M. purified ComGC, and S. E., P. S., C. D. L., K. T., and U. A. determined the NMR structure and dynamics. T. B. performed class averages on pili. S. M., S. E., M. S. A., and B. H. N. wrote the paper. All authors reviewed the results and approved the final version of the manuscript.

## Supplementary Material

Supplemental Data

## Footnotes

3 The abbreviations used are:

CSP: competence stimulating peptide
BACTH: bacterial adenylate cyclase two-hybrid
hetNOE: heteronuclear ^1^H-^15^N-NOE
O/N: overnight
RDC: residual dipolar coupling
Ni-NTA: nickel-nitrilotriacetic acid.
