# Supplementary material for: Structure of the competence pilus major pilin ComGC in Streptococcus pneumoniae
Source: J Biol Chem. 2017 Jun 28;292(34):14134–46. doi: 10.1074/jbc.M117.787671 (PMC5572924; doi:10.1074/jbc.M117.787671)
Supplement: Supplemental Data [file supp_292_34_14134__index.html]

Structure of the competence pilus major pilin ComGC in Streptococcus pneumoniae — Solution structure of ComGC from S. pneumoniae — Supplemental Data 

# Structure of the competence pilus major pilin ComGC in *Streptococcus pneumoniae*

## Supplemental Data

- Supplemental Data (.pdf, 898 KB) - Supplemental data including tables, figures and figure legends.
