# Supplementary material for: Structure of the competence pilus major pilin ComGC in Streptococcus pneumoniae
Source: J Biol Chem. 2017 Jun 28;292(34):14134–46. doi: 10.1074/jbc.M117.787671 (PMC5572924; doi:10.1074/jbc.M117.787671)
Supplement: Supplemental Data [file 10.1074_M117.787671_jbc.M117.787671-1.pdf]

Table S1. *S. pneumoniae* strains used in this study

Strain	Description	Source/reference
T4	Type 4 strain TIGR4	Tettelin *et al.* (2001)
T4R	*cps4A::cm* (Cm^R) in T4	Fernebro *et al.* (2004)
R6	R6 strain	kind gift of R. Hakenbeck
T4RΔ*rrgA-srtD*	T4R lacking the rlrA operon encoded pilus	Barocchi *et al.* (2005)
T4Δ*comGC*	*comGC::erm* (Em^R)	Balaban *et al.* (2014)

Em^R erythromycin-resistant, Cm^R chloramphenicol-resistant.

Table S2. Primers used in this study

Name	Sequence	Description
94F	cgcgcggccgcTTTACGAATAGATAAGTATGATT	Cloning *pilD* into pJWV25
96F	cgcgcggccgcTCAGACAAGCAGTAGCTTACCAAAAA	Cloning *pilD* into pJWV25
1F	cgccatatgATGAAAAAAATGATGACATTCTT	Cloning *comGC* in pACYCDuet-1
2R	cgcctcgagTTAATCATTGACTTTACGATTTGC	Cloning *comGC* in pACYCDuet-1
149F	cgcggatccCTTTACATTGGTGGAGATGTTGGTG	Cloning *comGC* in pKT25 and pUT18C
133R	cgcgaattcTTAATCATTGACTTTACGATTTGC	Cloning *comGC* in pKT25 and pUT18C
173F	cgctctagaGTTCACCTTACTGGAAATCATGGTG	Cloning *pulG* in pKT25 and pUT18C
174R	cgcgaattcCTATTTCTTCCCGATCGTCCAGTTGCCG	Cloning *pulG* in pKT25 and pUT18C

Restriction sites are underlined.

Table S3. Plasmids used in this study

Name	Description	Source/reference
pJWV25-*pilD*	Vector expressing PilD	This study
pACYCDuet-1-*comGC*	Vector expressing full-length ComGC	This study
pKT25	BACTH vector designed to express a protein fused to the C-terminal end of T25	Karimova *et al.* (2001)
pUT18C	BACTH vector designed to express a protein fused to the C-terminal end of T18	Karimova *et al.* (2001)
pKT25-zip	Vector expressing the leucine zipper of GCN4 fused to T25 (control plasmid)	Karimova *et al.* (2001)<br>Karimova *et al.* (2001)
pUT18C-zip	Vector expressing the leucine zipper of GCN4 fused to T18 (control plasmid)	This study
pKT25-*comGC*	vector expressing T25-ComGC	This study
pUT18C-*comGC*	vector expressing T18-ComGC	This study
pKT25-*pulG*	vector expressing T25-PulG	This study
pUT18C-*pulG*	vector expressing T18-PulG	This study

FIGURE LEGEND SUPPLEMENTARY FIGURES:

SUPPLEMENTARY FIGURE S1: Experimentally obtained ComGC secondary structure. A, Secondary chemical shift analysis. Here the obtained Cα , Cβ, C' and Hα chemical shifts are compared to random coil chemical shifts. For α-helical segments the δΔ values for Cα and C' are positive whereas Hα and Cβ are negative (red). δΔ values close to zero indicate no or very little secondary structure (grey). B, Agadir α-helix prediction. α2 and α3 are predicted but not α1-C. C, Jpred prediction of expected secondary structure probabilities. Here, all three helices are predicted for the sequence. Predicted helical regions with low confidence scores are displayed in light red.

SUPPLEMENTARY FIGURE S2: 15N-NOE strips for residues 41-69. The strips of the ~14 first N-terminal residues largely lack inter-residual peaks which is a consequence of faster dynamics and a general lack of structure. In contrast, residues 58-69 display many inter-residual NOE peaks and NH-NH couplings as would be expected from α-helical secondary structure.

SUPPLEMENTARY FIGURE S3: Sequence variation in ComGC. Unrooted phylogenetic tree of 24 *S. pneumoniae* strains clustered according to their ComGC sequence and multiple sequence alignment using Geneious software (1).

FIGURE S1.

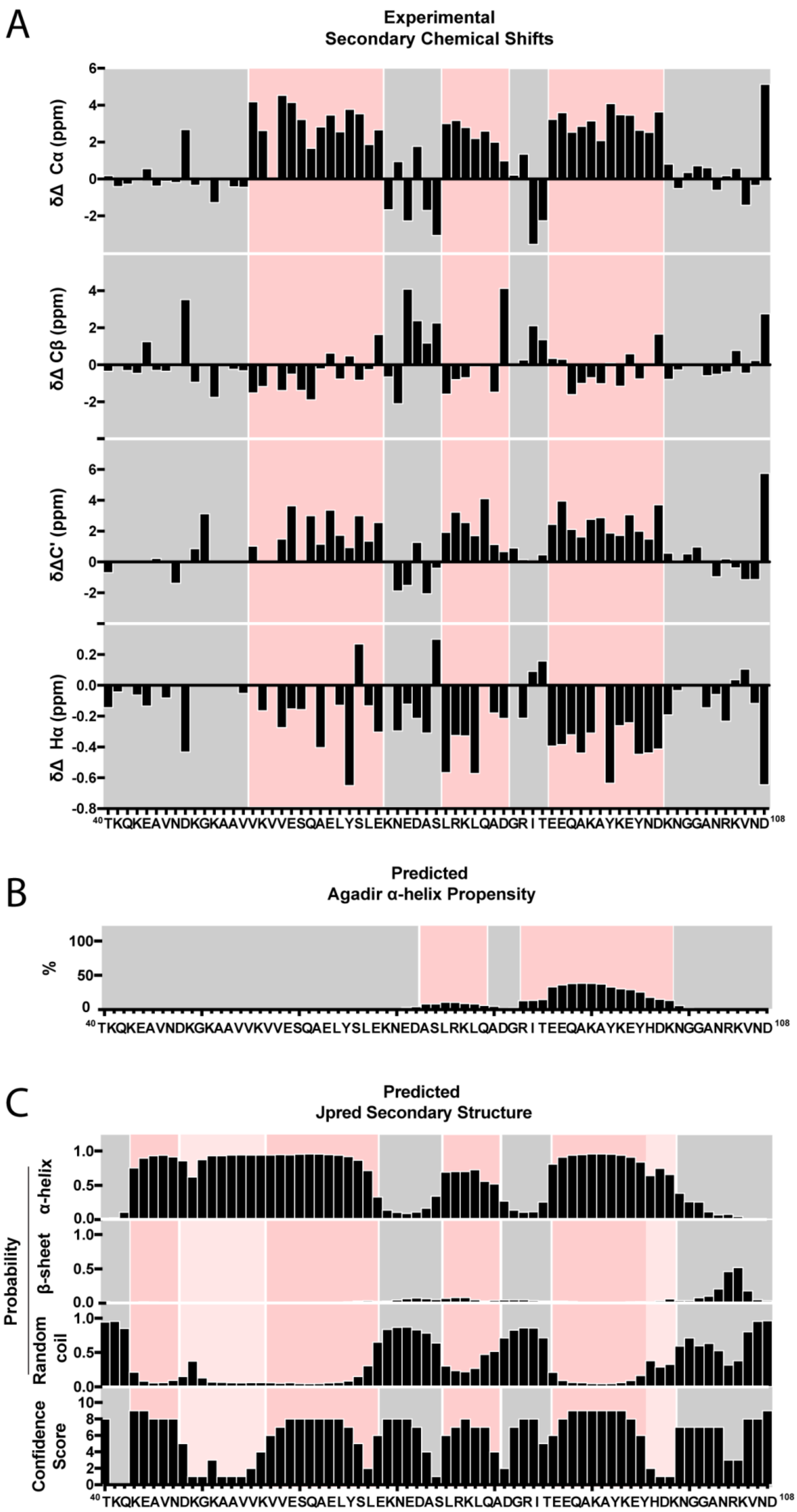
A
Experimental
Secondary Chemical Shifts
δΔ Cα (ppm)
δΔ Cβ (ppm)
δΔC' (ppm)
δΔ Hα (ppm)
40 TKQKEAVNDKGKAAVVKVVESQAELYSLEKNEDASLRKLQADGRI TEEQAKAYKEYNDKNGGANRKVND 108
B
Predicted
Agadir α-helix Propensity
%
40 TKQKEAVNDKGKAAVVKVVESQAELYSLEKNEDASLRKLQADGRI TEEQAKAYKEYHDKNGGANRKVND 108
C
Predicted
Jpred Secondary Structure
Probability
α-helix
β-sheet
Random coil
Confidence Score
40 TKQKEAVNDKGKAAVVKVVESQAELYSLEKNEDASLRKLQADGRI TEEQAKAYKEYHDKNGGANRKVND 108

FIGURE S2.

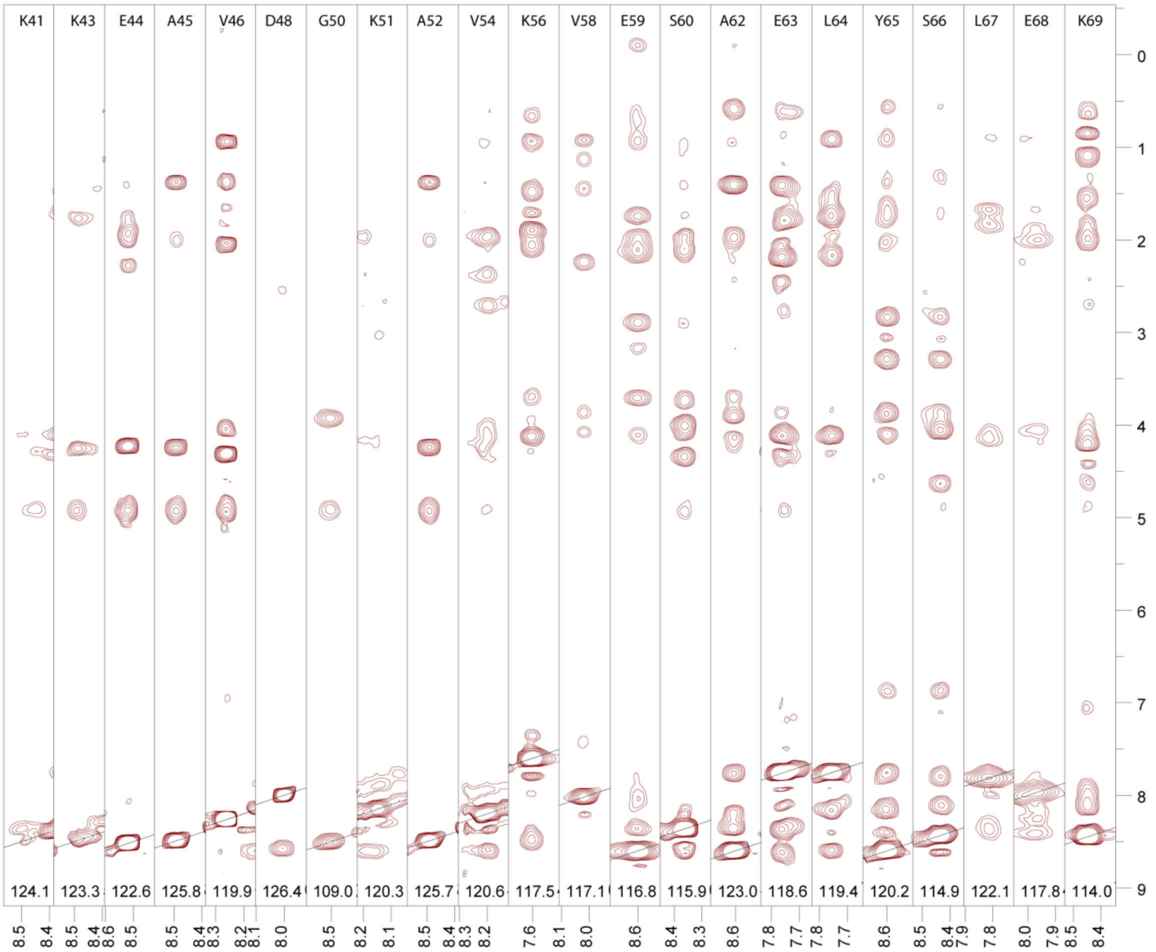
K41
K43
E44
A45
V46
D48
G50
K51
A52
V54
K56
V58
E59
S60
A62
E63
L64
Y65
S66
L67
E68
K69
124.1
123.3
122.6
125.8
119.9
126.4
109.0
120.3
125.7
120.6
117.5
117.1
116.8
115.9
123.0
118.6
119.4
120.2
114.9
122.1
117.8
114.0
0
1
2
3
4
5
6
7
8
9

FIGURE S3.

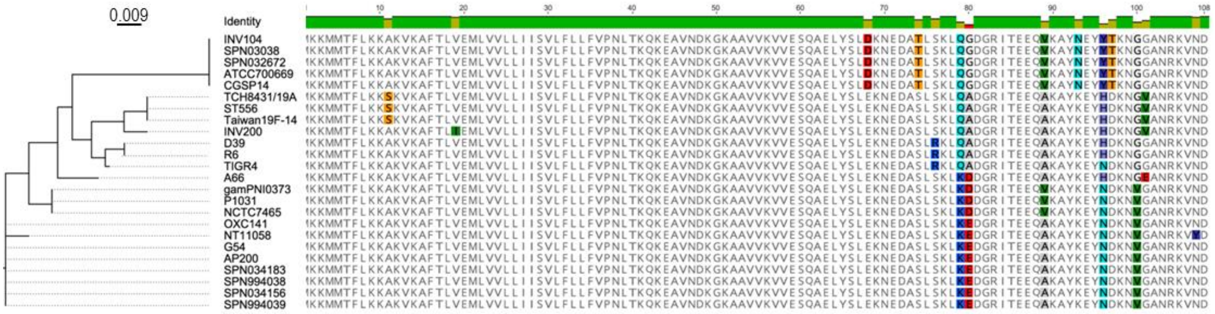

REFERENCES

1. Kearse, M., Moir, R., Wilson, A., Stones-Havas, S., Cheung, M., Sturrock, S., Buxton, S., Cooper, A., Markowitz, S., Duran, C., Thierer, T., Ashton, B., Meintjes, P., and Drummond, A. (2012) Geneious Basic: an integrated and extendable desktop software platform for the organization and analysis of sequence data. *Bioinformatics* **28**, 1647-1649
